# Detection of parathyroid adenomas with multiphase 4DCT: towards a true four-dimensional technique

**DOI:** 10.1186/s12880-021-00597-1

**Published:** 2021-04-07

**Authors:** Steven Raeymaeckers, Yannick De Brucker, Tim Vanderhasselt, Nico Buls, Johan De Mey

**Affiliations:** 1 Department of Radiology, Universitair Ziekenhuis Brussel, Laarbeeklaan 101, 1090 Jette, Belgium; 2 Medical Physics, Vrije Universiteit Brussel, Laarbeeklaan 103, 1090 Jette, Belgium; 3 Radiology, Vrije Universiteit Brussel, Laarbeeklaan 103, 1090 Jette, Belgium

**Keywords:** Parathyroid, Hyperparathyroidism, Endocrine disorders, 4DCT, CT dose reduction

## Abstract

**Background:**

Four-dimensional computed tomography (4DCT) is a commonly performed examination in the management of primary hyperparathyroidism, combining three-dimensional imaging with enhancement over time as the fourth dimension. We propose a novel technique consisting of 16 different contrast phases instead of three or four different phases. The main aim of this study was to ascertain whether this protocol allows the detection of parathyroid adenomas within dose limits. Our secondary aim was to examine the enhancement of parathyroid lesions over time.

**Methods:**

For this prospective study, we included 15 patients with primary hyperparathyroidism and a positive ultrasound prior to surgery. We performed 4DCT with 16 different phases: an unenhanced phase followed by 11 consecutive arterial phases and 4 venous phases. Continuous axial scanning centered on the thyroid was performed over a fixed 8 cm or 16 cm coverage volume after the start of contrast administration.

**Results:**

In all patients, an enlarged parathyroid lesion was demonstrated, and the mean lesion size was 13.6 mm. The mean peak arterial enhancement for parathyroid lesions was 384 Hounsfield units (HU) compared to 333 HU for the normal thyroid. No significant difference could be found. The time to peak (TTP) was significantly earlier for parathyroid adenomas than for normal thyroid tissue: 30.8 s versus 32.3 s (*p* value 0.008). The mean slope of increase (MSI) of the enhancement curve was significantly steeper than that of normal thyroid tissue: 29.8% versus 22.2% (*p* value 0.012). The mean dose length product was 890.7 mGy cm with a calculated effective dose of 6.7 mSv.

**Conclusion:**

Our 4DCT protocol may allow better visualization of the pattern of enhancement of parathyroid lesions, as enhancement over time curves can be drawn. In this way, wash-in and wash-out of contrast in suspected lesions can be readily demonstrated. Motion artifacts are less problematic as multiple phases are available. Exposure to our proposed 4DCT technique is comparable to that for classic helical 4DCT. Careful selection of parameters (lowering kV and SNR) can help to further reduce the dose.

## Background

Primary hyperparathyroidism (PHPT) is a common endocrine disease and is defined as hypercalcemia with increased or inappropriately normal plasma parathyroid hormone (PTH). PHPT is usually caused by benign proliferation of chief cells in a single enlarged parathyroid gland, and sometimes multiple glands can be affected. Rarely, an association can be found with multiple endocrine neoplasia (MEN) syndrome [1]. The incidence of PHPT in the US is estimated at 34–120 cases per 100,000/y (women) and 13–36 cases per 100,000/y (men); women are three- to fourfold more affected than men. There is also a higher incidence in Blacks and elderly individuals (70–79y) [2]. A clinical presentation of elevated calcium levels is associated with severe osteodynia, pruritus, pathologic fractures, muscle weakness and more vague symptoms such as memory loss, concentration difficulties or depression [3]. Hypercalcemia is most commonly seen asymptomatically; however, even these “asymptomatic” patients can present skeletal deterioration and other subclinical manifestations (osteoporosis, hypercalciuria, clinically silent vertebral fractures, nephrolithiasis) [4].

In the case of an asymptomatic patient with PHPT over the age of 50 without end-organ complications, conservative treatment can be assumed [5, 6]. The only cure for the disease is surgery, with resection of the affected gland(s). Bilateral exploration of the neck is the historical standard for treatment. In recent decades, however, this procedure has been abandoned in favor of a minimally invasive surgical approach made possible only by more effective means of preoperative imaging combined with the development of rapid parathyroid hormone determination techniques [7].

Many different imaging modalities exist. The most accessible diagnostic technique is ultrasound: it is widely available at low cost and presents no adverse effects [8]. The sensitivity is very operator-dependent but can be as high as 84% in the hands of an experienced ultrasonographer [9]. Color Doppler can be used to differentiate parathyroid lesions from other cervical masses, such as lymph nodes and thyroid nodules [10]. Very small lesions (< 5 mm) can be difficult to detect. False-negative results can occur, especially in cases of ectopic glands or in the presence of a large thyroid goiter [11].

Scintigraphy has the highest reported sensitivity: 88–90% and higher when combined with SPECT/SPECT-CT, the latter providing useful anatomical detail [12]. This technique is the method of choice when ectopic localization is suspected or in patients who have undergone prior neck surgery [13].

MRI also allows for the evaluation of parathyroid disease but remains a far less available modality. Due to the lack of ionizing radiation, it can be used without hesitation to detect ectopic glands. On a 1.5 T system, the reported sensitivity of this technique is 80% [14–16].

Computed tomography (CT) after administration of contrast combines anatomic and functional information. By evaluation of enhancement, abnormal parathyroid glands can be detected with a sensitivity of 85.7% [17, 18]. It is considered a useful technique in cases of ectopic glands or in cases of persistence/recurrence after initial surgery. The term four-dimensional computed tomography (4DCT) first appeared in the literature in 2000, when Ichikawa et al. [19] suggested a four-dimensional technique with the addition of contrast dynamics to three-dimensional hepatic tumor imaging. The concept of 4DCT for the detection of parathyroid adenomas was coined in 2006 by Rodgers et al. [20]. These authors used a three-phasic approach with an unenhanced phase and arterial and venous scanning phases. Many different study protocols have been suggested by different authors over the past 15 years. An optimal timing of the arterial phase is important for detecting parathyroid adenomas: this way, wash-in of contrast can be visualized, which is suggestive of parathyroid adenomas. We reviewed the literature to determine the ideal timing of this arterial phase.

We performed a comprehensive search of the literature in the PubMed and MEDLINE electronic databases up until May 2020 using MeSH terms “4DCT” and “parathyroid” and identified 51 articles citing defining parameters of the scanning protocol. These articles are listed in Table 1 [17, 18, 20–68]. Taking duplicates into account, we identified 38 unique scanning protocols.

**Table 1:** Overview of the timing of the different scanning phases obtained via 4DCT protocols for primary hyperparathyroidism in the literature

Reference	Authors	Year published	NECT	Tracking	Contrast phase timing	Number of phases	Arterial phase	Venous phase	Delayed phase	Very delayed phase
[21]	Vijayasarathi et al	2020	Yes	Yes	Relative	3	25–30 s	60–80 s	–	–
[22]	Wojtczak et al	2020	Yes	No	Relative	4	25–30 s	55–60 s	85–90 s	–
[23]	Acar et al	2020	Yes	No	Absolute	4	25 s	40 s	80 s	–
[24]	Zafereo et al	2019	Yes	No	Relative	3	25–30 s	55–60 s	–	–
			Yes	No	Relative	3	25–30 s	–	85–90 s	–
[25]	Kedarisetty et al	2019	Yes	No	Absolute	3	25 s	–	80 s	–
[26]	Yeh et al	2019	Yes	No	Relative	3	30 s	60 s	–	–
[27]	Amadou et al	2019	Yes	No	Absolute	3	–	45 s	70 s	–
[28]	Vu et al	2019	Yes	No	Absolute	4	25 s	55 s	85 s	–
[29]	Binks et al	2019	Yes	No	Absolute	3	25–30 s	45–50 s	–	–
[30]	Cunha‐Bezerra et al	2018	Yes	Yes	Relative	4	18–25 s	48–55 s	?	–
[31]	Tian et al	2018	Yes	Yes	Relative	3	Aorta: 150 HU	30 s later	–	–
[32]	Christakis et al	2017	Yes	No	Absolute	4	25 s	?	?	–
[33]	Morón et al	2017	Yes	No	Absolute	2	25 s	–	–	–
[34]	Goroshi et al	2017	Yes	No	Absolute	4	20 s	60 s	90 s	–
[35]	Taywade et al	2017	Yes	No	Absolute	3	25 s	–	80 s	–
[36]	Zeina et al	2017	Yes	No	Absolute	4	25 s	60 s	90 s	–
[37]	Sho et al	2016	Yes	No	Relative	3	25 s	55 s	–	–
[38]	Fitzgerald et al	2017	Yes	No	Absolute	3	30 s	–	90 s	–
[39]	Rameau et al	2017	Yes	No	Relative	3	25 s	55 s	–	–
[40]	Ramirez et al	2016	Yes	No	Absolute	4	25 s	55 s	85 s	–
			Yes	No	Absolute	2	25 s	–	–	–
[41]	Forghani et al	2016	Yes	No	Absolute	4	25 s	55 s	85 s	–
[42]	Lee et al	2016	Yes	No	Absolute	4	30 s	60 s	90 s	–
[43]	Hinson et al	2015	Yes	No	Absolute	4	30 s	60 s	90 s	–
[44]	Bahl et al	2015	Yes	No	Absolute	3	25 s	–	80 s	–
[45]	Boury et al	2015	Yes	No	Absolute	3	–	45 s	70 s	–
[46]	Hoang et al	2015	Yes	No	Absolute	3	25 s	–	80 s	–
[47]	Lundstroem et al	2016	Yes	No	Absolute	5	22 s	52 s	82 s	122 s
[48]	Seeliger et al	2015	Yes	No	Absolute	4	25 s	50 s	80 s	–
[49]	Cham et al	2015	Yes	No	Relative	3	25–34 s	55–64 s	–	–
[50]	Day et al	2015	Yes	No	Absolute	4	30 s	60 s	90 s	–
[51]	Campbell et al	2015	Yes	No	Absolute	2	–	50 s	–	–
[52]	Sepahdari et al	2015	Yes	No	Absolute	3	25 s	55 s	–	–
			Yes	No	Absolute	3	25 s	–	80 s	–
[53]	Suh et al	2015	Yes	No	Absolute	4	30 s	60 s	90 s	–
[54]	Ginsburg et al	2015	Yes	No	Absolute	4	25 s	55 s	85 s	–
[55]	Raghavan et al	2014	Yes	No	Absolute	4	25 s	50 s	80 s	–
[56]	Brown et al	2015	Yes	No	Absolute	3	34 s	68 s	–	–
[18]	Hoang et al	2014	Yes	No	Absolute	3	25 s	–	80 s	–
[57]	Hunter et al	2014	Yes	No	Relative	3	30 s	60 s	–	–
[58]	Bahl et al	2014	No	No	Absolute	2	20 s	70 s	–	–
			Yes	No	Absolute	3	25 s	–	80 s	–
[59]	Kelly et al	2014	Yes	No	Relative	4	30 s	45 s	90 s	–
			Yes	No	Relative	3	30 s	45 s	–	–
[60]	Sepahdari et al	2013	Yes	No	Relative	3	25–34 s	55–84 s	–	–
[61]	Hunter et al	2012	Yes	No	Relative	4	25 s	70–73 s	–	130–133 s
[62]	Mahajan et al	2012	Yes	No	Absolute	4	30 s	60 s	90 s	–
[63]	Gafton et al	2012	No	No	Absolute	2	25 s	–	80 s	–
[64]	Kutler et al	2011	Yes	No	Absolute	2	–	50 s	–	–
[65]	Eichhorn-Wharry et al	2011	No	No	?	2	18 s	Immediately	–	–
			No	No	?	2	22 s (> 55 y)	Immediately	–	–
[17]	Starker et al	2011	Yes	No	Absolute	4	30 s	60 s	–	120 s
[66]	Beland et al	2011	Yes	No	Absolute	4	30 s	60 s	90 s	–
[67]	Lubitz et al	2010	Yes	No	Relative	4	30 s	60 s	–	105 s
[68]	Mortenson et al	2008	Yes	No	Relative	4	25 s	55 s	85 s	–
[20]	Rodgers et al	2006	Yes	No	?	3	25 s	?	–	–

The timing of the different scanning phases in the literature varied: with the exception of four studies, all cited authors chose to obtain an arterial phase. The arterial images in the literature were obtained as early as 18 s and as late as 34 s after administration of contrast. In the case of 25 different studies, the arterial images were obtained 25 s after contrast administration; in 13 studies, the authors proposed scanning after 30 s. Five other authors stated that the arterial images were obtained by scanning after 25–30 s. Forty-two authors then performed arterial scanning after 25 to 30 s (identical protocols included).

The main aim of this study was to ascertain whether we could create a 4DCT protocol allowing for the detection of parathyroid adenomas using multiple added contrast phases within dose limits. Our secondary aim was to examine the enhancement of parathyroid lesions over time, determining the maximum peak enhancement of parathyroid lesions and thus suggesting an optimal window for the arterial phase(s).

## Methods

For this prospective study, we included 15 patients with primary hyperparathyroidism (i.e., an elevated serum level of calcium and raised levels of parathyroid hormone) and a positive ultrasound prior to surgery. This study was approved by the medical ethics committee of our hospital. All the patients were informed about the nature of the procedure as well as the risks involved (radiation, administration of iodinated contrast) and signed an approved informed consent form prior to the examination.

Patients under the legal age (18 y) were excluded. Patients who had undergone a prior surgery of the thyroid or parathyroid were also excluded. All the patients had undergone a prior US examination, as this is the standard of care for detecting parathyroid adenomas in our hospital.

Scanning was performed on a 256-slice Revolution CT (GE Healthcare, Waukesha, Wisconsin, USA). The patients received a venous catheter placed in a cubital vein that was checked for patency. Their arms were placed in a neutral position alongside the body. The patient’s head was fixed in a head cradle. The sensation of contrast administration was explained to the patients, and they were instructed not to move and to avoid swallowing. Continuous axial scanning centered on the thyroid was performed over a fixed 8 cm coverage volume (100 kVp, SmartmA 10–480 mA, thickness 0.625 mm, 0.5 s rotation scanning time). Wide beam axial scanning was chosen over helical scanning to limit the dose [69]. For the last three patients, we widened the scanning range from an 8 cm volume to a 16 cm volume, covering a larger view of the upper mediastinum.

**Fig. 1:**
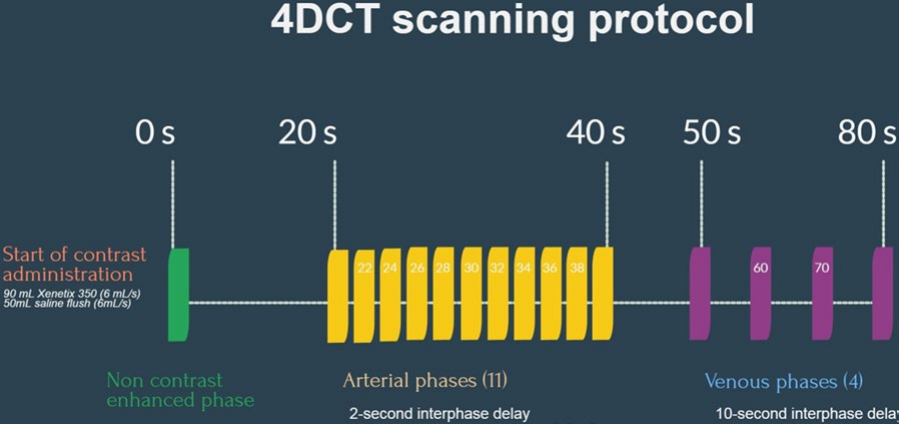
Schematic overview of the different phases in our 4DCT protocol

First, we obtain a nonenhanced scan (NECT). Simultaneously, contrast administration was initiated: a bolus of 90 mL Xenetix 350 mg I/mL was injected at 6 mL/s followed by a 50 mL saline flush (6 mL/s). After a delay of 20 s, 11 subsequent phases with a 2-s interphase delay were obtained (arterial phases). With a 10-s interphase delay, 4 more phases were obtained (venous phases). A schematic overview of this protocol is provided in Fig. 1.

The images were reviewed on an Advantage Workstation VolumeShare 7 (GE Healthcare, Waukesha, Wisconsin, USA). The different scan phases were deformably registered, after which the registered image data could be analyzed on a voxel-by-voxel basis, thereby retaining spatial information for the analysis.

The data were analyzed and interpreted by two senior members of the staff with 11 and 12 years of experience in the field of neuroradiology as well as head and neck radiology and perfusion imaging experience, respectively.

The following parameters were considered in the normal thyroid, parathyroid and lymph nodes. The maximum peak enhancement (HUmax) is defined as the maximum concentration of contrast agent over time and was measured in the region of interest. It is expressed in Hounsfield units (HU). The time to peak (TTP) is defined as the time at which HUmax is reached. It is expressed in seconds (s). The mean slope of increase (MSI) corresponds to the steepness of the enhancement curve of a given region of interest. It is expressed in percent (%). An overview of these different values is provided in Table 2.

**Table 2:** Overview of adenoma size

	Size (mm)	HUmax lesion (HU)	HUmax thyroid (HU)	HUmax lymph (HU)	TTP lesion (s)	TTP thyroid (s)	MSI lesion (%)	MSI thyroid (%)	DLP (mGy.cm)	E (mSv)
1	7.5	421	276	82	26.2	28.2	40.9	26.1	1122.3	8.4
2	14	505	279	92	30.8	33.6	40.5	12.5	703.5	5.3
3	12	264	326	115	31.4	34.7	18.8	9.9	643.3	4.8
4	9	381	318	93	35.6	39.6	21.7	11.9	1064.2	8.0
5	6	315	290	88	37.3	36.4	21.6	10.8	1083.4	8.1
6	35	454	321	97	32.0	32.4	34.5	18.1	742.5	5.6
7	7	372	491	123	29.7	32.7	32.9	24.2	199.7	1.5
8	11	487	334	101	28.0	28.9	40.9	23.9	656.1	4.9
9	12	366	317	92	28.2	26.2	36.1	37.6	552.9	4.1
10	15	518	342	84	26.6	27.9	40.9	40.5	827.2	6.2
11	5	302	390	109	26.9	28.4	16.7	38.6	962.9	7.2
12	9	327	384	104	28.1	29.2	34.9	33.0	181.8	1.4
13	22	392	355	112	30.4	30.2	25.9	19.6	631.5	4.7
14	30	262	246	102	42.2	44.9	8.4	5.4	1992.9	14.9
15	9	391	318	138	29.2	31.0	31.8	20.4	1996.1	15.0
Mean value	13.6	383.8	332.5	102.1	30.8*	32.3*	29.8**	22.2**	890.7	6.7

HUmax values are given for both parathyroid and thyroid tissue as well as lymph nodes. Time to peak (TTP) and mean slope of increase (MSI) of parathyroid and thyroid tissue. Dose length product (DLP) and effective dose (E)

*p-value 0.008

**p-value 0.012

Statistical analyses were performed using SPSS software ver. 23.0 (IBM, Armonk, NY, USA). For this small dataset, we assumed the absence of normality and symmetry. The Wilcoxon signed rank test was used to evaluate differences between values. Statistical significance was set at *p* < 0.020.

For the evaluation of the radiation dose, we considered the dose length product (DLP) and the effective dose (E). The DLP is a technical dose descriptor expressed in mGy.cm that represents the total radiation output of the scan. The effective dose, expressed in mSv, represents the total body dose and was calculated following current ICRP-103 guidelines [70] by using CT patient dosimetry software (CT-Expo v1.7.1, G Stamm and H Nagel, Hann-over) that considers all technical acquisition parameters of the individual scans, including the scan range. A conversion factor of 0.0075 was used.

## Results

The mean age of the included patients was 61 years. Six patients were male; nine patients were female. In all the patients, a single enlarged parathyroid could be detected, coinciding with ultrasound findings and surgical localization. The mean lesion size was 13.6 mm. The mean peak arterial enhancement for parathyroid lesions was 384 HU compared to 333 HU for the normal thyroid. No significant difference could be found between these values.

**Fig. 2:**
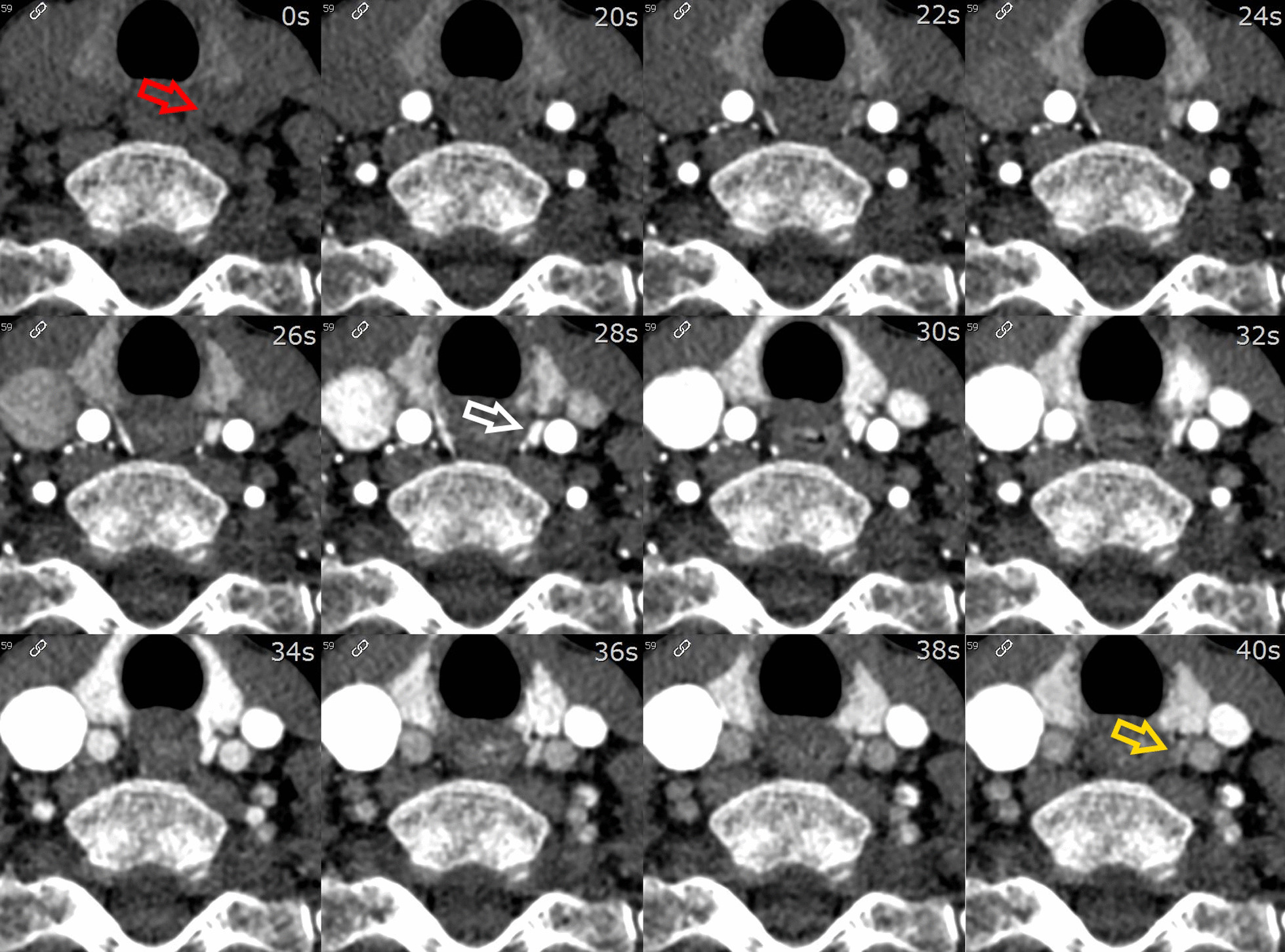
Axial images at the level of the thyroid at different time intervals. Scans at 0–20–22–24–26–28–30–32–34–36–38–40 s after administration of contrast, left to right from the top. Suspected parathyroid adenoma posterior from the left thyroid lobe in close relation to the common carotid artery (arrows). Note the lower enhancement on NECT (red arrow), higher enhancement compared with the thyroid in the arterial phase (white arrow) and the wash-out of contrast in the early venous phase (yellow arrow)

**Fig. 3:**
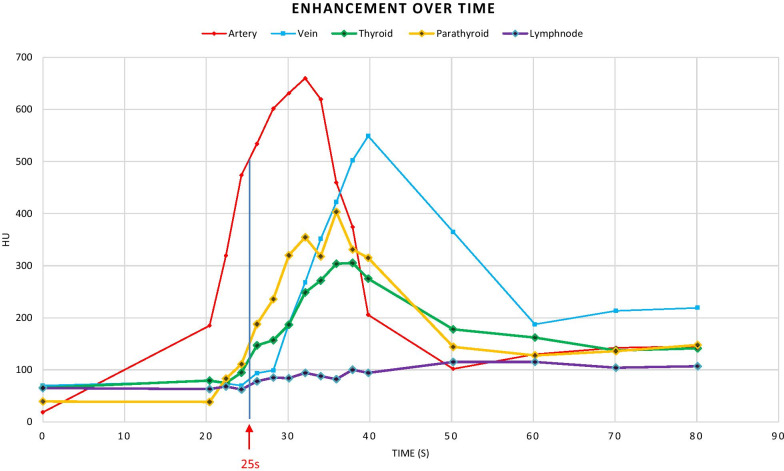
Enhancement (Y-axis) over time (X-axis) curve. The curve for the suspected parathyroid (yellow) is steeper than the curve for the thyroid (green), and the peaks for the artery (red) and vein (blue) are higher in comparison. An artifact due to motion is observed at 34 s with suboptimal HU measurement of the small parathyroid lesion. The graph also demonstrates venous wash-out of the suspected parathyroid (50–70 s). Additionally, note the lack of prominent difference in enhancement between the adenoma and the thyroid at 25 s after administration of contrast. The purple curve indicates slow continuous enhancement of a lymph node

The different phases were reviewed side-by-side, as demonstrated in Fig. 2. In this example, we identified a parathyroid adenoma posterior to the left thyroid lobe in close relation to the common carotid artery. The different phases were also reviewed in absolute relation to their timing. This allowed us to plot the various phases on a curve depicting enhancement over time, as demonstrated in Fig. 3. In the example shown, arterial wash-in and venous wash-out of a suspected parathyroid adenoma were demonstrated with ease. Motion artifacts due to swallowing were present on a single phase in this specific example, affecting the curve of the small parathyroid lesion (34 s after administration of contrast).

Eleven out of 15 detected adenomas demonstrated a higher peak enhancement in the arterial phase than that in normal thyroid tissue (arterial wash-in). Four out of 15 adenomas had a slightly lower peak enhancement in the arterial phase than that in normal thyroid tissue. These four adenomas showed a lower enhancement than the thyroid in the later venous phases (venous wash-out). The enhancement pattern of the lymph nodes differed from the parathyroid and thyroid tissue with lower maximum enhancement (mean HUmax 102 HU). These lesions did not demonstrate a significant wash-in or wash-out of contrast.

The mean TTP for parathyroid adenomas was calculated at 30.8 s (interval 26–42 s). The mean TTP for normal thyroid tissue was calculated at 32.3 s (interval 26–45 s). The TTP for parathyroid adenomas was observed significantly earlier (*p*-value 0.008). The MSI was significantly steeper for the adenomas (29.8% vs 22.2%, *p*-value 0.012).

The mean dose length product was 890.7 mGy cm with a calculated effective dose of 6.7 mSv. The mean dose length product for the scans with an 8 cm volume was 728.3 mGy.cm with a calculated effective dose of 5.5 mSv. The mean dose length product for the three scans with a 16 cm volume was 1540.2 mGy cm with a calculated effective dose of 11.6 mSv.

## Discussion

In all the patients, a single enlarged parathyroid could be detected, coinciding with ultrasound findings and surgical localization. By drawing enhancement over time curves, we easily evaluated the pattern of enhancement of a region of interest over time. In this way, wash-in and wash-out of contrast was readily demonstrated. Motion artifacts (due to swallowing, for example) can affect the measurement in the ROI of small lesions, as we demonstrated in one example (Fig. 3). If this effect would have been present on a single and only arterial phase, this might have resulted in missing the lesion, as the difference in enhancement between the lesion and the normal thyroid did decrease in the affected series. Since we possessed other phases for this case, this problem was at least partially overcome.

We found a statistically significant difference in the TTP and MSI between parathyroid and thyroid tissue, with parathyroid adenomas showing a steeper curve and becoming enhanced on average 1.5 s earlier. Since the interval for the TTP for parathyroid adenomas in our study varied between 26 and 42 s after contrast administration, arterial enhancement was less conspicuous with a single arterial phase 25 s after contrast administration (the most common protocol found in the literature).

Cervical lymph nodes are a known mimicker of parathyroid adenomas. Based on their slow and continuous enhancement, these structures can be readily differentiated from parathyroid adenomas. In the case of cervical lymph node metastasis, we could expect an alteration of this enhancement pattern. In a patient presenting with PHPT symptoms, lymph node metastasis would have to be considered an incidentaloma and thus proven to be rare. Localized spread from a parathyroid carcinoma could be considered an associated possibility; however, this disease is also increasingly rare (reported incidence 0.5 to 5% of primary hyperparathyroidism cases) [71]. Another known mimicker of a parathyroid adenoma is ectopic thyroid tissue. It can be suggested that this ectopic thyroid tissue would behave in a similar way to normal thyroid tissue and thus differ from parathyroid adenomas. As we did not come across a relevant case, this remains speculation.

The effective dose associated with our proposed 4DCT protocol has a mean value of 6.7 mSv and can be as low as 1.4 mSv. The effective dose of 4DCT protocols in the literature is situated between 10.4 mSv and 13.8 mSv. The effective dose of scintigraphy is estimated at 7.8 mSv (99mTc-sestamibi-SPECT) or 18.4 mSv (hybrid sestamibi-SPECT) [43, 62]. The last three patients were scanned with a 16 cm volume. The calculated effective dose for these three cases was calculated at 11.5 mSv, still within the dose limits for traditional helical 4DCT, as referenced in the literature [62]. We then managed to define the parameters for a dynamic 4DCT examination within an acceptable exposure range. Since our proposed technique relies more on contrast enhancement of suspected lesions than anatomical detail, further dose reduction can be achieved by lowering the signal-to-noise ratio (SNR).

This study has several limitations. First there is the small sample size of the study. It is, however, difficult to include patients when an existing and approved standard of care technique such as ultrasound produces excellent results. Our study is also prone to selection bias since we only included patients with positive ultrasound findings who were eligible for surgery. Lesions in patients when surgery is not considered may be smaller and thus more difficult to detect. Comparing the suspected parathyroid lesion to thyroid tissue may also prove problematic in cases of thyroid disease such as thyroiditis with associated diffuse inflammation of the thyroid gland and in cases of (suspicious) thyroid nodules. This may especially prove true in cases of intrathyroidal parathyroid adenomas. Last, we admit that our initial scanning protocol may not have been ideal for the detection of ectopic glands, as an 8 cm volume is small. A 16 cm volume is still within the possibilities of axial scanning and can then include the upper mediastinal structures; however, the dose will be higher, as stated above. Careful selection of parameters (lowering kV and SNR) can help to overcome this issue.

## Conclusion

Our 4DCT protocol may allow better visualization of the pattern of enhancement of parathyroid lesions, as enhancement over time curves can be drawn. In this way, wash-in and wash-out of contrast in suspected lesions can be readily demonstrated. Since the interval for the TTP of parathyroid adenomas in our study varied between 26 and 42 s after contrast administration, arterial enhancement of the lesion could prove less conspicuous when scanning 25 s after contrast administration, the most common practice in the literature. Motion artifacts are less problematic as multiple phases are available. TTP was found to be significantly earlier for parathyroid adenomas than for normal thyroid tissue, and the MSI was significantly steeper. Normal lymph nodes can be readily differentiated from parathyroid adenomas due to their slow and continuous enhancement. Exposure to our proposed 4DCT technique is comparable to that for classic helical 4DCT. Careful selection of parameters (lowering kV and SNR) can help to further reduce the dose.

## Data Availability

The data analyzed during the current study are included within the article. The different sets of CT images are not publicly available due to medical confidentiality.

## Abbreviations

4DCT: 4-Dimensional computed tomography
DLP: Dose length product
HU: Hounsfield units
MRI: Magnetic resonance imaging
MSI: Mean slope of increase
NECT: Non enhanced computed tomography
PHPT: Primary hyperparathyroidism
ROI: Region of interest
SNR: Signal to noise ratio
SPECT: Single-photon emission computed tomography
TTP: Time to peak
